# Longitudinal Associations Between Physical Activity Behavior and Structural Brain MRI Features After Stroke: A Sub-Study From the Nor-COAST Project

**DOI:** 10.1177/15459683251399125

**Published:** 2026-01-09

**Authors:** Geske Luzum, Eva B. Aamodt, Heather Allore, Dag Alnæs, Mona K. Beyer, Ann-Marie G. de Lange, Ingvild Saltvedt, Till Schellhorn, Lars T. Westlye, Torunn Askim, Asta K. Håberg

**Affiliations:** 1 Department of Neuromedicine and Movement Science, NTNU - Norwegian University of Science and Technology, Trondheim, Norway; 2 Division of Radiology and Nuclear Medicine, Oslo University Hospital, Oslo, Norway; 3 School of Medicine, Yale University, New Haven, USA; 4 Department of Psychology, University of Oslo, Oslo, Norway; 5 Center for Precision Psychiatry, Division of Mental Health and Addiction, Oslo University Hospital, Oslo, Norway; 6 Institute of Clinical Medicine, University of Oslo, Oslo, Norway; 7 Department of Psychiatry, University of Oxford, Oxford, UK; 8 Department of Geriatric Medicine, Clinic of Medicine, St. Olavs Hospital, Trondheim University Hospital, Trondheim, Norway

**Keywords:** brain maintenance, neuroimaging, brain age, physical activity, secondary prevention

## Abstract

**Background:**

Post-stroke physical activity (PA) behavior may partly explain inter-individual differences in cortical and sub-cortical brain volumes and brain age estimates.

**Objective:**

To investigate longitudinal associations of post-stroke PA behavior with structural brain MRI features.

**Methods:**

Data were from a multicenter longitudinal cohort study. PA estimates were based on accelerometer measurements. Separate linear mixed models assessed average daily step count at 18 and 36 months, and longitudinal PA trajectory groups as measured at 3, 18, and 36 months after stroke, as primary and secondary exposures. Dependent variables included brain age gap (BAG), representing the discrepancy between brain MRI predicted age and chronological age, and MRI-based cortical, hippocampal, and thalamic volumes at 18- and 36 months post-stroke. Models accounted for age, sex, education, stroke severity, intracranial volume, and MRI scanner.

**Results:**

We included 146 participants (age, mean [SD]: 70.3 [11.1]; 45.7% female) with predominantly mild strokes. Every +1000 steps/day were associated with −1.15 (95% CI: −1.76 to −0.53) lower BAG, 2.63 mL (95% CI: 0.31-5.00) larger cortical volume, and 0.07 mL (95% CI: 0.03-0.11) larger hippocampal volume. The association between step/day and thalamic volume was curvilinear, with the largest volumes observed at 4700 steps/day. Out of 4 PA trajectory groups, participants in the most active group had −7.44 years (95% CI: −2.86 to −12.01) lower BAG and 0.90 mL (95% CI: 1.48-0.33) larger thalamic volumes than the least active group.

**Conclusions:**

Higher PA levels post-stroke were associated with larger brain volumes and younger-appearing brains.

## Graphical abstract

**Graphical Abstract:**
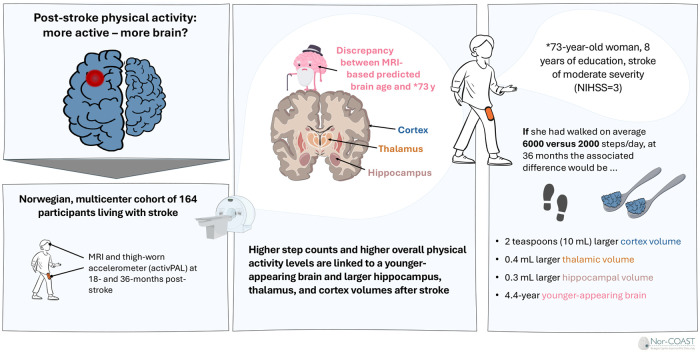

## Introduction

After a stroke, neural cell death, including axonal degeneration, may accelerate brain aging and brain atrophy.^1^ Cortical,^1,2^ hippocampal, and thalamic^3,4^ volume loss is common in both the early and chronic phases post-stroke.^5^ However, the rate of brain aging and atrophy following stroke show substantial individual and regional differences, for example, depending on patient age and overall health, as well as stroke severity and location.^1,2,6-8^ Moreover, modifiable life style and health risk factors such as high blood pressure,^9^ a history of smoking, unhealthy diet, and other factors have also been shown to be associated with neurodegeneration and clinical outcomes post-stroke.^10^

Two closely related concepts, brain reserve and brain maintenance, are often used in the context of “resilience” to age- and disease-related structural changes in the brain.^11^ Brain reserve refers to the neurobiological capital at a given timepoint, which allows some individuals to better cope with brain aging and pathology before clinical symptoms emerge. Brain maintenance refers to the progression of age-related brain changes and pathology over time, with better brain maintenance reducing such changes.^11^ As brain atrophy, that is, reduced brain reserve, precedes and predicts cognitive decline after stroke,^5,8,12^ identifying modifiable factors influencing brain reserve and/or maintenance could help identify people with stroke at increased risk of cognitive decline and provide knowledge on possible preventive interventions.

Physical activity (PA), a modifiable life-style factor and potential non-pharmacological modifiable target, has been suggested to improve brain maintenance in brain regions vulnerable to dementia in older adults after stroke; thus, potentially improve recovery and provide secondary prevention.^13^ Human and animal stroke studies suggest that PA promotes neuronal changes by enhancing synaptic plasticity and connectivity.^14^ No previous study has investigated longitudinal associations between habitual PA and brain maintenance following stroke. In healthy adults and older adults, PA and exercise have been linked to brain maintenance, mitigating or limiting atrophy in cortical and subcortical areas, with the most consistent findings in the hippocampus.^13,15,16^ Further, brain age gap (BAG),^17^ a proxy for global brain health, has been associated with PA behavior,^18,19^ with lower BAG, indicating better brain reserve. BAG represents the discrepancy between a person’s predicted age from brain magnetic resonance imaging (MRI) scans and chronological age.^17^

To understand the association between post-stroke PA and brain maintenance, this study examined longitudinal associations between objective, sensor-based PA behavior and structural brain MRI features in individuals with stroke, from the sub-acute to the chronic phase. We hypothesized that

1. Higher daily step counts at 18-, and 36-months post-stroke were associated with lower BAG and larger cortical, hippocampal, and thalamic volumes at 18- and 36-months post-stroke.
2. Individuals with PA behaviors characterized by multiple interruptions of daily sedentary behavior in combination with high engagement in light and moderate PA from 3 to 36 months post-stroke had lower BAG, and larger cortical, hippocampal, and thalamic volumes at 18- and 36-months post-stroke compared to their peers with less PA engagement.

## Methods

### Study Design and Participants

**Figure 1.**
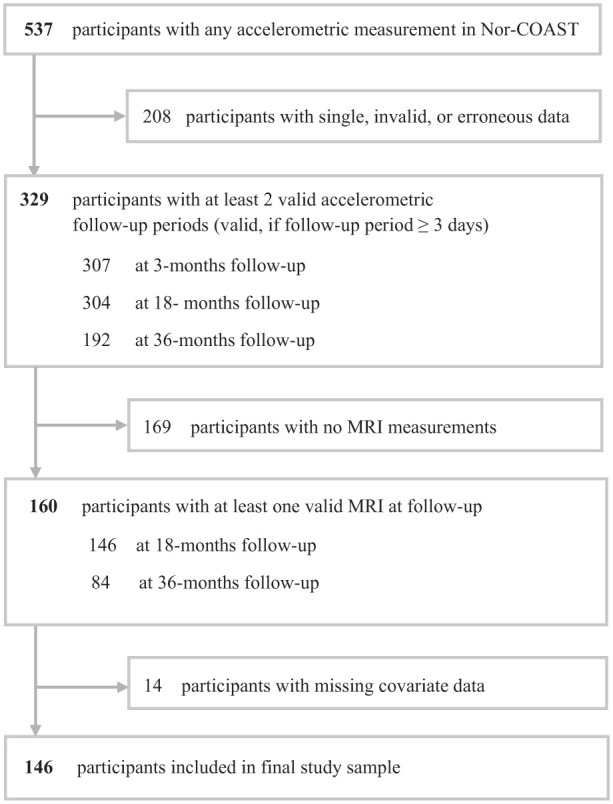
Flow of participants. The figure shows the final number of participants after applying the inclusion criteria. Abbreviations: Nor-COAST, Norwegian Cognitive Impairment After Stroke Study; MRI, magnetic resonance imaging.

This study was a longitudinal, multi-center cohort study, based on data from the Norwegian Cognitive Impairment After Stroke Study (Nor-COAST).^20^ The research was approved by the ethics committee REK Nord (REK number: 2015/171; 2017/1415) and complied with the Declaration of Helsinki. Between March 2015 and March 2017, individuals with acute stroke admitted to 1 of 5 participating hospitals were consecutively recruited within 1 week after symptom onset. Eligible individuals provided written informed consent before inclusion. Those unable to consent were included if their next of kin did not decline participation. Stroke was diagnosed according to the World Health Organization; as “a focal (or global) neurological impairment of sudden onset and lasting more than 24 hours (or leading to death) of presumed vascular origin”.^21^ Individuals were eligible to participate in Nor-COAST if they were fluent in a Scandinavian language, >18 years of age, and had an expected survival of >3 months based on assessment by experienced stroke physicians. For this study, individuals were required to have at least 3 consecutive days of accelerometer data at a minimum of 2 follow-up time points (3, 18, and/or 36 months), and at least 1 MRI with successful FreeSurfer anatomical segmentation at 18 and/or 36 months. In Nor-COAST, 160 participants met these criteria. Participants with missing covariate data (see below) were subsequently excluded giving a final sample of 146 (27.2%) participants (Figure 1).

### Clinical Data

Baseline assessment was performed at discharge or within the 7th day of the hospital stay. Sociodemographic and health information, including stroke characteristics, were from electronic medical records and interviews with participants and/or next of kin. Global premorbid function was assessed with the modified Rankin Scale (mRS).^22^ The etiology of ischemic strokes was classified with the Trial of Org 10172 in Acute Stroke Treatment (TOAST) classification and stroke severity evaluated 1 day after symptom debut with the National Institutes of Health Stroke Scale, obtained at day 1 after admission to hospital (NIHSS-1), ranging from 0 to 42 on a continuous scale.^23,24^ Global cognitive function post-stroke was obtained at the 3-month follow-up using the Montreal Cognitive Assessment (MoCA) with scores ranging from 0 to 30. One point was added to the MoCA score if participants had ≤12 years of education.^25^ Global disability was assessed 3 months after stroke with mRS.^26^

### Covariates

Supplementary Figure S1 visualizes *a priori* assumptions about the association of potentially confounding variables with PA behavior and the brain MRI features. In addition, intracranial volume and MRI scanners were used as covariates in analyses with brain volumes.

### MRI Acquisition and Analysis

As part of a sub-study in Nor-COAST, participants were invited to undergo brain MRI during their initial hospital stay (baseline), and at 18- and 36-month follow-up. Additional inclusion criteria for MRI were modified Rankin scale <5 before the stroke and the ability to cooperate during an MRI scan. MRI exclusion criteria were standard MRI contraindications. According to the ethical approval, reasons why patients declined participation were not recorded.^12^

This study used harmonized 3D T1-weighted MRI.^5,27^ MRI data at 18, and 36 months were processed in the longitudinal stream of FreeSurfer 6.0.1 (http://surfer.nmr.mgh.harvard.edu/), which included brain extraction, intensity normalization, tissue segmentation, and surface reconstruction,^28^ to estimate the total intracranial volume, cortex, hippocampus, and thalamus volumes. Left and right cortex, hippocampus, and thalamus volumes were combined to a total volume.

To calculate BAG, we applied a previously trained age prediction model,^27^ using eXtreme Gradient Boosting (XGBoost)^29^ in Python 3. Input features included estimates of cortical thickness, surface area, and gray and white matter volumes based on the Desikan-Killiany atlas^30^ and subcortical FreeSurfer segmentations. Independent samples of healthy controls from the Cambridge Center for Aging and Neuroscience^31^ and StrokeMRI^18^ (n = 934, age range = 18-88 years, mean = 55.8, SD = 17.4) were used to train the model. For hyperparameter tuning and model evaluation, nested cross-validation with 5 inner and 10 outer folds was employed using scikit-learn.^32^ To address age-related bias in the predictions, the main effect of age, age,^2^ and sex were regressed out using linear regression modeling.^33^ BAG represents the discrepancy between an individual’s predicted and chronological age, with lower values indicating a younger-appearing brain.

### Accelerometry

The widely used definition of PA as any bodily movement produced by skeletal muscles that requires energy expenditure, including activities at work, home, travel, and recreation was adopted in this work, with our measurements limited to upright behaviors such as standing and walking, as captured by the thigh-worn, 3-axial accelerometer activPAL (PAL Technologies Ltd., Glasgow, United Kingdom).^34,35^ Participants were instructed to wear the device, attached to the front of their unaffected thigh, for 7 days following a 24-hour wearing protocol at 3, 18, and 36 months follow-ups. Data were retrieved and converted to spreadsheets using the pre-classified algorithm VANE from PALanalysis. Visual data inspection ensured that erroneous days and non-wearing periods were removed. To retrieve estimations of daily PA levels accumulated during waking periods only, we used an automated algorithm separating waking time from time in bed.^36^ The sum of accumulated steps per measurement day during estimated wake periods at 18 and 36 months was extracted, and the daily average step count calculated as mean per measurement period. Details on data collection and quality control are reported elsewhere.^37^

### The PA Trajectory Groups

In a previous study, multi-trajectory modeling (see Supplemental Material) was used to identify groups with different PA profiles based on co-occurrence of light PA (any standing or walking activities below 3 Metabolic Equivalents of Task, METs), moderate PA (any walking ≥3 to <6 METs), and the number of sit-to-stand transitions over time (3, 18, and 36 months follow-up). Four different PA groups emerged.^37^

*Frequent and high group*: ~22% of participants engaged in an average of >1 hour of moderate PA over time, performed most, yet decreasing light PA, and had stable frequent sit-to-stand transitions. This most active group was used as a reference group.

*Frequent and intermediate group*: ~15% of the participants had comparable duration of light PA and moderate PA as the infrequent and intermediate group but engaged in more sit-to-stand transitions. This group remained stable over time in duration estimates and only reduced the number of transitions.

*Infrequent and intermediate group*: ~26% of the participants spent almost twice as much time in moderate PA and light PA as the infrequent and low group but had similar number of sit-to-stand transitions as the former group. Moderate PA and sit-to-stand transitions decreased over time.

*Infrequent and low group*: the ~37% of our participants were characterized by overall low PA and modest linear decline over time in the 3 PA dimensions.

### Statistical Analysis

In separate analyses, BAG, hippocampus, thalamus, and cortex volume (18- and 36 months post-stroke) were outcomes in linear mixed effect models (LMEs) with participant as a random effect to address heterogeneity. The mean number of steps per measurement period was entered as an independent fixed effect, along with covariates time, sex, age, education, and stroke severity. For all volumetric outcome variables (cortex, thalamus, and hippocampus) the models were further adjusted for estimated intracranial volume and MRI scanner (Supplementary Figure S1). An autoregressive covariance structure accounted for within-participant correlation. To evaluate for curvilinear effects of average step count and potential time interactions, we added a quadratic term of step count and a time interaction with step count, which were dropped if insignificant. To facilitate interpretation, we calculated and plotted margins averaging over all covariates.

We repeated the above-described LME modeling for the second hypothesis, using PA group as primary exposure. Because the categorical PA groups represent PA behavior phenotypes from 3 to 36 months post-stroke, they remain constant over time. Therefore, we specified an independent correlation structure and omitted quadratic time effects or time interactions.

The Holm-Bonferroni method was used to correct for multiple comparisons.^38^ Concerns of potential selection bias due to attrition were addressed by a sensitivity analysis using inverse probability weighting (Supplementary Table S1). To assess robustness, all original LMEs were conducted in (1) a simple (unadjusted) version, (2) a propensity score weighted version using the same model specification (full adjustment), and (3) a propensity score weighted, simple (unadjusted) version. In addition, we used multiple regression models as specified in Supplementary Table S7 to assess whether conclusions from the longitudinal LMEs would hold when changing time lags by entering only 36-month MRI data; and used linear mixed models with the additional covariates home care services (yes/no), marital status, and symptoms of anxiety and depression.

Lastly, we calculated E-values as post-estimation sensitivity analysis for unmeasured confounding in observational studies using the methodology proposed by VanderWeele and Ding.^39^ E-values assess how robust an association is to potential unmeasured or uncontrolled confounders. The interpretation of E-values depends on context and the covariates for which adjustments were made. Higher E-values indicate that unmeasured confounding is less likely to negate an observed treatment effect.^39^

Descriptive data were presented as mean ± SD and N (%) for categorical variables unless otherwise stated. Quality control and statistical analyses were performed using STATA Version 17.0 software (StataCorp, College Station, TX, USA).

## Results

Study participants (n = 146, age: 70.3 [11.1], 45.7% women) had primarily ischemic (95.2%) mild strokes (NIHSS-1: 2.83 [4.69]) and had a median MoCA score of 26 (75th and 25th percentiles: 24-28) at 3 months. Details on participant characteristics, including the longitudinal estimates of PA variables and MRI measures are presented in Table 1 (and in Supplementary Tables S2 and S3).

**Table 1.** Participant Characteristics.

Baseline	N	Values
Female, n (%)	146	60 (45.7)
Age, yrs, mean (SD)	146	70.28 (11.14)
Education, yrs., mean (SD)	146	12.90 (3.85)
BMI, mean (SD)	146	26.38 (4.18)
NIHSS-1, mean (SD)	146	2.83 (4.69)
Lesion type, n (%)	146	
Ischemic stroke		139 (95.21)
Hemorrhagic stroke		7 (4.79)
TOAST, n (%)	146	
Arteriosclerosis		12 (8.2)
Cardiac emboli		37 (25.3)
Small vessel disease		34 (23.3)
Other		2 (1.4)
Unknown		61 (41.8)
Comorbidity (CCS), mean (SD)	146	3.61 (1.79)
Pre-stroke (mRS), mean (SD)		0.62 (0.79)
3 months		
mRS, mean (SD)	146	1.38 (0.91)
MoCA, mean (SD)	142	25.63 (3.14)
Median (75th and 25th percentiles)		26 (24 to 28)
18 months		
Average daily step count, mean (SD)	142	2565 (1583)
Cortex volume (mL), mean (SD)	135	433.69 (48.31)
Thalamus volume (mL), mean (SD)	135	12.66 (1.71)
Hippocampus volume (mL), mean (SD)	135	7.20 (1.08)
BAG (y), mean (SD)	135	− 0.74 (9.69)
36 months		
Average daily step count, mean (SD)	94	2970 (1751)
Cortex volume (mL), mean (SD)	81	430.28 (43.67)
Thalamus volume (mL), mean (SD)	81	12.43 (1.54)
Hippocampus volume (mL), mean (SD)	81	7.14 (1.09)
BAG (y), mean (SD)	81	−2.37 (9.80)

Abbreviations: BMI, body mass index; CCS, Charlson Comorbidity Scale; mRS, modified Rankin Scale; MoCA, Montreal Cognitive Assessment; BAG, brain age gap.

National Institutes of Health Stroke Scale, measured 1 day after symptom debut (NIHSS-1, range 0-42), The Trial of Org 10,172 in Acute Stroke Treatment (TOAST).

### Association of Average Step Count and Structural Brain MRI Features

**Figure 2.**
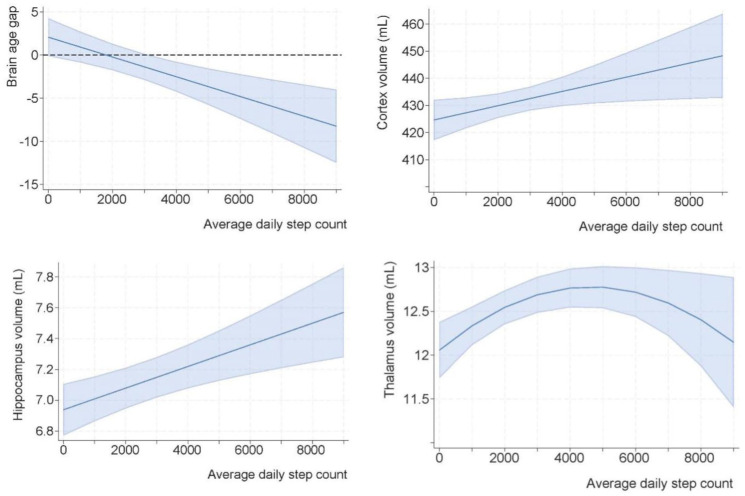
Margin plots of the association of longitudinal step count and structural brain MRI features post-stroke. We present the results of Table 2 as margin plots, with 1 graph per model. To facilitate interpretation, the thalamic model visualizes the quadratic term (curvilinear association), while a more complex model including the time interaction is presented in Supplementary Figure S2. The dark blue lines represent the best-fit curves describing the exposure-outcomes associations, whereas the light blue areas show the estimated 95% confidence intervals. As a practical example, a 73-year-old woman, with 8 years of education who had a stroke of moderate severity (NIHSS = 3) and who walked on average 2000 steps/day (in this study, this is above the lower 25th percentile) would have an estimated BAG of 0.08 (95% CI −2.86 to 3.02) 3 years after the stroke. However, if the same woman walked on average 6000 steps/day the predicted margins of BAG would be −4.50 (95% CI: −8.28 to −0.72) 3 years after the stroke.

We found a significant association between average daily step count and brain MRI features post-stroke across time (Table 2). Figure 2 visualizes how average daily step counts were associated with individual differences in BAG, cortex, hippocampus, and thalamus volumes at 18- and 36-months post-stroke. Results revealed a negative linear association between step count and BAG after adjusting for covariates. Each 1000-step increase in average daily step count was associated with a −1.15 (95% CI: −1.77 to −0.53) lower BAG. The E-value for this association was 5.12 (lower 95% CI: 2.61).

**Table 2.** Linear Mixed Effect Models Association of Average Daily Step Count and Structural Brain MRI Features (N = 146).

Dependent variable	Effect	Coefficient	95% CI	*P*-value
BAG (years)	Steps	−1.15	−1.77 to −0.53	<.001*
Cortex (mL)	Steps	2.63	0.31 to 4.95	.027*
Hippocampus (mL)	Steps	0.07	0.03 to 0.11	.001*
Thalamus (mL)	Steps	0.28	0.10 to 0.45	<.001*
	Steps^2^	−0.03	−0.06 to −0.01	.01*
	Time × steps	0.10	0.05 to 0.16	<.001*

LME estimates are adjusted for sex, age, education, time of measurement, and NIHSS-1; for the volumetric analyses, we additionally adjusted for magnetic resonance imaging (MRI) scanner and total estimated intracranial volume. The hippocampus and thalamus estimates represent the sum of estimated volumes of left and right ventricles accordingly. Fixed effects (steps) refer to the average daily step count measured at 3-, 18-, and 36-months post-stroke, where 1 unit change equals 1000 steps. The volumetric estimates are given in milliliters (mL). *P*-values marked with a star (*) indicate significance at a .05 level, after Holm-Bonferroni adjustment. See supplemental Figure S3 for model comparisons with and without weighting and adjustment.

We uncovered a positive linear association between average daily step count and cortex and hippocampus volumes. For each +1000-step increase in average daily step count, cortex volumes were estimated to be 2.63 mL (95% CI: 0.31-5.00) higher, corresponding to an average 0.61% larger cortex volume at 18- and 36-months. Each +1000 increased steps was associated with larger hippocampal volumes by 0.07 mL (95% CI: 0.03-0.11 mL), that is, on average 0.98% greater hippocampal volume at 18 and 36 months. The E-values for cortex and hippocampus models were 1.05 (lower 95% CI: 1.02) and 1.01 (lower 95% CI: 1.01), respectively.

There was no interaction between daily step count and time since stroke on the cortex and hippocampus volumes. However, the association between average daily step count and thalamus volume was curvilinear and characterized by an interaction with time since stroke (supplementary Figure S2). A positive association was estimated for those with the lowest step count; and increasing thalamus volumes, peaking around 4700 steps/day. Hence, a person with ~1000 steps/day was estimated to have 0.28mL (2.23%) higher thalamus volume compared to someone with close to zero steps/day. Conversely, an individual with ~5000 steps/day had only <0.01% larger thalamus volume compared to a person with 6000 steps/day. The E-value for that association (based on a linear term) was 1.02 (lower 95% CI: 1.01). All investigated associations between step count and brain MRI metrics were significant after Holm-Bonferroni corrections. The estimates were similar after inverse probability weighting (Supplementary Figure S3) and additional model adjustments (marital status, home-care services, symptoms of anxiety and depression), reflecting robust results.

### Association of PA Group Membership and Structural Brain MRI Features

**Figure 3.**
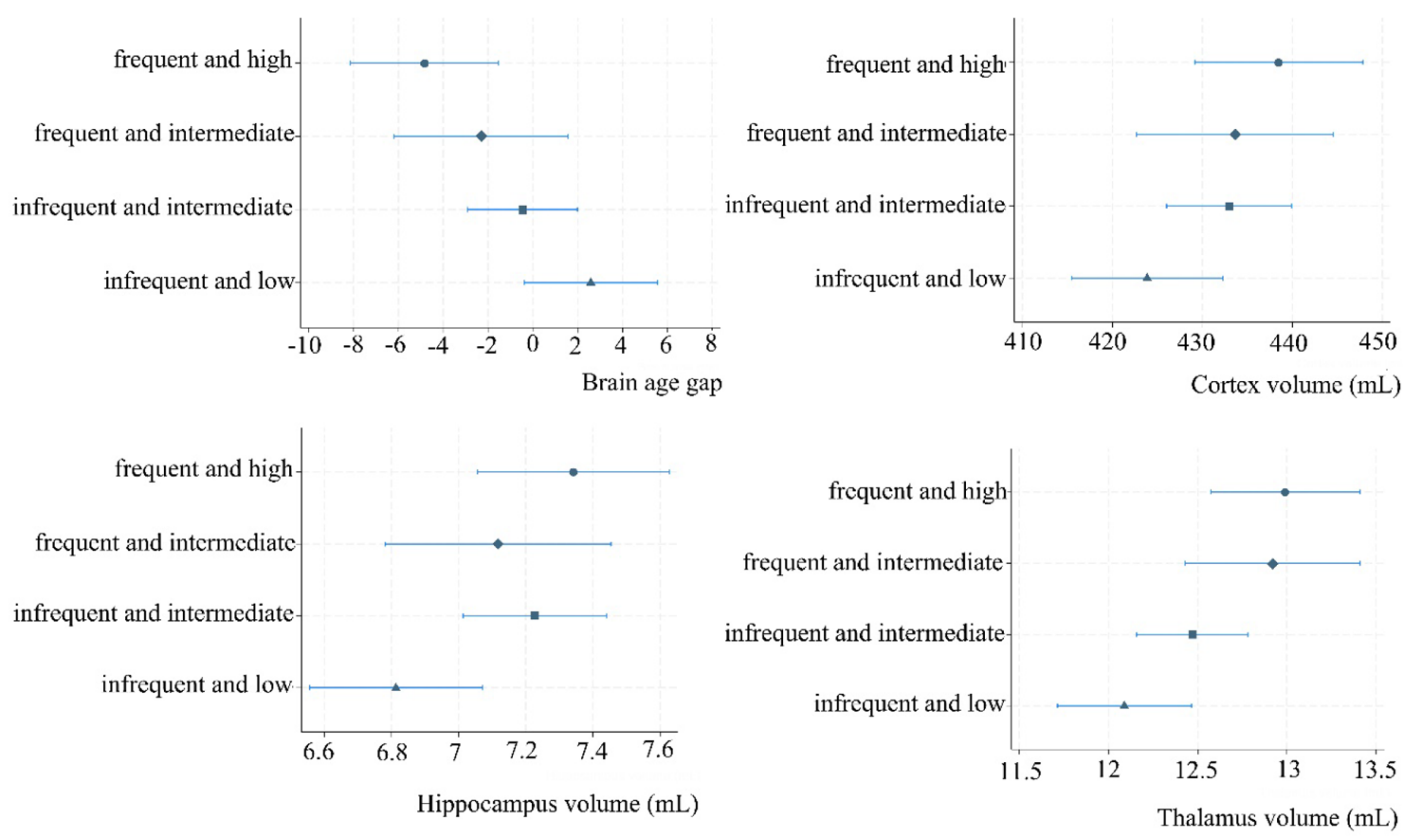
Margin plots of the association of PA trajectory group membership and selected brain MRI features. Abbreviations: MRI, magnetic resonance imaging; mL, milliliter.

The PA groups and brain MRI features revealed significant differences between the least active “infrequent and low” and the reference group on BAG, cortex-, hippocampus-, and thalamus volumes (Table 3 and Figure 3). The “infrequent and low” group had a significantly higher BAG and smaller cortical, hippocampal, and thalamic volumes compared to the reference group. We further observed a difference in BAG between the “infrequent and intermediate” and the reference group. However, after correction for multiple comparisons, only BAG and thalamus volume in the “infrequent and low” group differed significantly from the reference group. Adjusted LME analyses revealed that the “infrequent and low” group had a higher BAG of 7.44 (95% CI: 2.86-12.01), and −0.90 (95% CI: −1.48 to −0.33) mL smaller thalamus volumes compared to the “frequent and high” reference group. No other significant differences in BAG, cortex, hippocampus, and thalamus volumes were present. The alternative models are presented in Supplementary Figure S4.

**Table 3.** Linear Mixed Effect Models Association of PA Trajectory Group Membership and Structural Brain MRI Features (N = 146).

Model (N = 146)	Explanatory variable	Coeff.	95% CI	*P*
BAG (years)	Ref: Frequent and high			
	Infrequent and low	7.44	2.86 to 12.01	0.001*
	Infrequent and intermediate	4.39	0.22 to 8.55	0.039
	Frequent and intermediate	2.54	−2.55 to 7.62	0.328
Cortex (mL)	Ref: Frequent and high			
	Infrequent and low	−14.62	−27.50 to −1.73	0.026
	Infrequent and intermediate	−5.50	−17.27 to 6.27	0.360
	Frequent and intermediate	−4.87	−19.22 to 9.49	0.506
Hippocampus (mL)	Ref: Frequent and high			
	Infrequent and low	−0.53	−0.92 to −0.13	0.009
	Infrequent and intermediate	−0.12	−0.48 to 0.25	0.533
	Frequent and intermediate	−0.22	−0.66 to 0.22	0.319
Thalamus (mL)	Ref: Frequent and high			
	Infrequent and low	−0.90	−1.48 to −0.33	0.002*
	Infrequent and intermediate	−0.52	−1.048 to 0.01	0.052
	Frequent and intermediate	−0.07	−0.72 to 0.57	0.826

Abbreviations: BAG, brain age gap; mL, milliliter.

Physical activity (PA) trajectory group membership is entered as an explanatory categorical variable. The “frequent and high” group is used as a reference group. Estimates are adjusted for sex, age, education, time of measurement, and stroke severity (NIHSS-1). We adjusted for magnetic resonance imaging (MRI) scanner and total estimated intracranial volume in volumetric analysis. The hippocampus and thalamus represent the sum of estimated volumes of the left and right ventricles accordingly. *P*-values marked with a star (*) indicate significance, at a .05 significance level, after the Holm-Bonferroni adjustment. The sensitivity analysis in Supplementary Figure S4 compares the results of alternative models of the association of PA trajectory group membership with structural brain MRI features suggesting a stepwise association between PA trajectory groups and brain age gap (BAG), cortex, hippocampus, and thalamus volumes.

The results of the post-hoc sensitivity analyses, including additional covariates, were similar to the models above, suggesting robust findings.

## Discussion

This study examined the longitudinal associations between objectively measured PA and structural brain MRI features in the chronic phase post-stroke. Our analyses revealed significant associations between higher daily step counts and a lower BAG, that is, a younger-appearing brain, and larger cortex, hippocampus, and thalamus volumes at 18 and 36 months. Next, individuals whose PA behavior was characterized by many interruptions of daily sedentary behavior in combination with high engagement in light and moderate PA from 3 to 36 months post-stroke, showed evidence of younger appearing brains (i.e., lower BAG), and higher thalamus volume at 18- and 36-months follow-up compared to less active peers. Overall, our analyses demonstrated a positive association between post-stroke PA and BAG, indicating that PA could decelerate brain aging in patients with strokes. Furthermore, slight differences in the results with cortex, hippocampus, and thalamus volumes as outcomes, suggested presence of anatomical differences in response to PA.

### PA and BAG

We found that with every +1000 step/day BAG was lower by −1.15 years (95% CI: −1.76 to −0.53). The PA group analysis further showed that individuals in the “infrequent and low” group had 7.44 (95% CI: 2.86-12.01) years higher BAG compared to the “frequent and high” group. The lack of significant differences in BAG across the other groups, despite substantial variation of average step counts across groups, suggest that PA as described by several dimensions (intensity, transitions, etc.) and their interplay may be important to consider. Our results in patients differed from those of a cross-sectional study in 122 healthy older adults that found no overall association between objectively measured step count and BAG, but 7-year younger-appearing brains per each +1000 step increase only among their female participants.^18^ While those sex-related differences might be explained by sex moderating the physiological response of PA in older adults,^40^ different findings may be attributed to differing neuroimaging modalities used to calculate BAG, as well as differing study populations. In summary, brain maintenance as proxied by BAG was better with more steps and in those belonging to the “frequent and high” PA group, for example, an overall higher PA volume.

### PA, Cortex, Hippocampus, and Thalamus Volume

Our analyses revealed a positive association between step count and cortical, thalamic, and hippocampal volumes in patients 18 and 36 months after a stroke and significantly larger (6.93%) thalamic volume in the most active PA group compared to the least active PA group. An explanation of why the associations between steps and cortical and subcortical regions were so consistent in contrast to cross-sectional studies in healthy adults^18,41-43^ may be due to the longitudinal approach and possibly more variance due to the clinical stroke cohort. Previously it was described that different study populations appear to have differential “sensitivity” to differing PA modalities.^44^ Similarly, the association between brain volumes and sensor-based PA has been shown to be more pronounced in older than younger individuals,^41^ and in individuals at genetic risk for dementia compared to those not at risk.^13,45^

### Brain Maintenance and PA Dosage

Our results demonstrated region-specific associations between PA and brain structures as evidenced by varying effect sizes and the shape of the associations. While associations between steps/day and structural brain MRI features were linear for BAG, cortex, and hippocampus, we found a curvilinear relationship between steps/day and thalamic volume with the highest thalamic volumes estimated at around 4700 steps/day. However, the observed linearity in the BAG, cortex, and hippocampus models must be interpreted within the observed data range (min to max: <1 to 8374 steps/day). Given the decreasing certainty of the models with increasing steps/day (wider 95% CI), the effect of steps may also have been curvilinear when steps/day exceed the observed range. Curvilinear (e.g., U or J-shaped) relationships between increasing step counts and energy expenditure on brain volumes have been described earlier in a cross-sectional population-based study.^44^ Potential explanations include increased inflammatory processes, particularly associated for instance with higher-intensity PA,^46^ or specific PA domains (work vs. leisure),^47^ which in turn may exacerbate brain health.^48^ Nevertheless, our findings indicated that any additional steps/day within the observed data range may help to maintain BAG, cortical, and hippocampal volumes, whereas the association between steps and thalamus volume suggested that potential benefits may plateau at higher step counts, or even be decreased. Further, the PA groups suggested a favorable association between higher PA volume (duration × frequency × x intensity) on brain maintenance, which should be investigated further.

All the participants in the most active “frequent and high” PA groups performed 150 minutes of moderate PA/week. We therefore believe this group is comparable to the control group of a 5-year exercise intervention study in older adults, who were supposed to follow the national PA guidelines .^49^ The control group exhibited less hippocampal and thalamic atrophy compared to participants in the high-intensity exercise group.^49^ These findings imply that moderate PA and free choice of the type of PA may be beneficial for brain maintenance, but needs to be replicated in the stroke population.

More research on behavior change techniques that could successfully and permanently increase habitual PA after stroke needs to be established, perhaps using feedback on behavior and biofeedback.^50^ As the accuracy, usability, and accessibility of commercially available smart watches and phones increase, monitoring PA levels may be feasible among older adults.^51^

### Brain Maintenance and PA Timing

The insignificant time interactions in the BAG, cortex, and hippocampus models indicated that the associations between steps/day and these outcomes remained consistent at both 18- to 36-months post-stroke. Our findings align with a meta-analysis of RCTs showing that exercise introduced on average 2.62 years after a stroke had significant, moderate, and positive effects on cognition and was not superior to PA training introduced within 3 months post-stroke.^52^ Still, we found a positive interaction between steps/day and time in the thalamus model, implying time since stroke influencing this association. As a result, the threshold at which increasing steps/day was no longer associated with larger thalamus volume was reached at a lower steps/day at 18 months than at 36 months (Supplementary Figure S2). Summarized, the relationship between steps/day appeared to saturate and change over time for thalamus volume, while for BAG, cortex, and hippocampus volumes, more steps/day at any time following stroke showed consistent outcomes.

Among the strengths of this study were the longitudinal design, the comprehensive clinical assessment including longitudinal MRI data, and objectively measured PA behaviors. Most studies examining the effect of PA on brain structure rely on self-reported PA^13^ susceptible to social desirability and recall bias,^53^ that may increase with age and cognitive impairments. In contrast, wearable sensors can provide an objective and detailed assessment of PA behavior, enabling the quantification of multiple metrics across key PA dimensions (duration, frequency, intensity) and time. In this study we used PA groups to account for the interrelationships between PA dimensions, which do not operate independently but interact to shape an individual’s overall PA behavior. Higher levels in 1 dimension may coincide with low levels in another and potentially have counteracting effects on an outcome.^49,54,55^ Further, by recruiting participants from several stroke units across Norway, we were able to include a representative study sample of individuals with predominantly mild strokes (86.3% with NIHSS-1 score 0-5), who make up most people living with stroke.^56-58^

This cohort’s clinical characteristics and risk factors were like the Nor-COAST cohort apart from participants included in the study being more likely to have higher mRS and lower MoCA and SPPB scores at 3 months. Inverse probability weighting successfully removed differences in age, education, and stroke severity (Supplementary Table S4) and did not change the results notably. Lastly, the use of random effects and sensitivity analyses proved our findings to be robust and unlikely to be explained by unmeasured confounding. Particularly, the E-value of the step count and BAG indicated reasonably robust results considering the context and existing confounding control, while the E-value of the cortex and hippocampus models in combination with wide confidence intervals may suggest a more careful interpretation.

### Limitations

This study has limitations. First, it is important to consider the potential selection bias within this sub-sample of the Nor-COAST population when interpreting our findings. The requirement for participants to meet in-person for testing, combined with the MRI-specific inclusion criteria, may have excluded some frailer participants, as suggested by an overall mortality of 17.3% in the entire Nor-COAST after 18-months follow-up, but only 2.1% in our cohort. Second, by aggregating volumetric brain estimates across hemispheres, we did not account for the stroke lesion side in this study. This may have diluted our results, as cortical and subcortical atrophy in the ipsilesional hemisphere has been shown to be greater than in the contralesional hemisphere over time.^5,7^ Third, we acknowledge the limitation that comes with the pragmatic approach to temporality in the models using PA groups. Although regression models confirmed our findings in a smaller study sub-sample only using 36-month MRI data (see Supplementary Table S7), the longitudinal study design in combination with overlapping time lags of 28 individuals who contributed accelerometer data from 36 months to estimate PA groups and having/had a single MRI measurement from 18 months follow-up did not allow for causal inference. Fourth, the possibility of bi-directional associations should be acknowledged and investigated, as earlier suggested for the link between exercise and dementia risk.^59^ Fifth, findings should be interpreted in light of the interdependence between exposure and outcomes. Structural brain measures in later life reflect the cumulative effects of inherent predisposition and lifetime exposures, not only those occurring during the study period. For example, previous research reported that entering older age with high cardiorespiratory fitness is protective against structural brain changes.^49^ This suggests that individuals in this cohort with higher PA levels may have had greater brain volumes both prior to and during the study, and consequently showed better brain maintenance. It is also important to consider the multidependency of PA and structural brain outcomes. Despite adjustment for random effects and potential confounders, other factors such as social support or environmental factors may have influenced our findings. Sixth, although activPAL shows excellent agreement with direct observation for upright time, its accuracy in detecting steps is limited at very slow walking speeds,^35^ potentially leading to an underestimation of step count in stroke survivors. Further, energy expenditure estimates based on METs may underestimate PA intensity in stroke survivors due to lower mechanical efficiency and fitness levels, with mobility-related activities requiring up to twice the oxygen cost compared to healthy norms.^60^ Lastly, we acknowledge the possibility of participants altering their PA behavior as a response to accelerometric follow-up, also known as the Hawthorne effect.

## Conclusion

This study provided evidence of the association between PA behavior and brain maintenance proxied by BAG, cortex, thalamus, and hippocampus volume in a stroke cohort. To translate the findings of habitual PA to clinical practice recommendations, research on the association between PA and cognitive function, optimal PA dosage, and time of initiation is needed. However, knowledge gaps should not prevent the implementation of interventions targeting individuals at risk of inactivity after stroke. While randomized controlled trials are needed to establish causality, our results indicate that higher engagement in PA behavior post-stroke is reflected in larger brain volumes and younger appearing brains at 18 and 36 months post-stroke, even only including light- to moderate-intensity PA. Accordingly, our study strengthens the evidence for established guidelines recommending an active lifestyle, also in the long-term, following stroke.

## Supplemental Material

sj-docx-1-nnr-10.1177_15459683251399125 – Supplemental material for Longitudinal Associations Between Physical Activity Behavior and Structural Brain MRI Features After Stroke: A Sub-Study From the Nor-COAST Project Supplemental material, sj-docx-1-nnr-10.1177_15459683251399125 for Longitudinal Associations Between Physical Activity Behavior and Structural Brain MRI Features After Stroke: A Sub-Study From the Nor-COAST Project by Geske Luzum, Eva B. Aamodt, Heather Allore, Dag Alnæs, Mona K. Beyer, Ann-Marie G. de Lange, Ingvild Saltvedt, Till Schellhorn, Lars T. Westlye, Torunn Askim and Asta K. Håberg in Neurorehabilitation and Neural Repair
