# Supplementary material for: Longitudinal Associations Between Physical Activity Behavior and Structural Brain MRI Features After Stroke: A Sub-Study From the Nor-COAST Project
Source: Neurorehabil Neural Repair. 2026 Jan 9;40(1):75–87. doi: 10.1177/15459683251399125 (PMC12799801; doi:10.1177/15459683251399125)
Supplement: sj-docx-1-nnr-10.1177_15459683251399125 – Supplemental material for Longitudinal Associations Between Physical Activity Behavior and Structural Brain MRI Features After Stroke: A Sub-Study From the Nor-COAST Project [file sj-docx-1-nnr-10.1177_15459683251399125.docx]

# **Supplementary material**

MRI scanner

age

time since stroke

eTIV

education

stroke severity

PA behavior

structural brain MRI features

**Figure S1** Directed acyclic graph (DAG) of the longitudinal association of physical activity (PA) behavior and structural brain MRI features at 18 and 36 months post-stroke.

Age, sex, education, stroke severity, and time since stroke are presented as confounders. Time since stroke was further added as an interaction term theorized to influence the relationship between the independent variables (PA behavior) and the dependent variables (brain age gap, cortex, hippocampus, and thalamus volume). Estimated intracranial volume (eTIV) and Magnetic Resonance Imaging (MRI) scanners were added as covariates to the volumetric models of hippocampus volume, cortex volume, and thalamus volume, only.

sex

# **A note on multi-trajectory modelling**

Trajectory models use maximum likelihood estimation to approximate the likelihood of each individual belonging to a group. The method is designed to measure the linkages between the trajectories of multiple differing but related outcomes, such as PA dimensions (duration, frequency, intensity, type), allowing us to include several PA dimensions simultaneously and estimate longitudinal heterogeneity.(Nagin 2014) This study used accelerometer data from 3, 18, and 36 months to construct the four PA trajectories that may be interpreted as differing phenotypes of PA behavior post-stroke.

# **Inverse probability weighting**

We conducted a sensitivity analysis to reflect uncertainty and test the robustness of the tested association between physical activity and structural brain MRI features. All original LME models were conducted in 1) a simple (unadjusted) version, 2) a propensity score weighted version using the same model specification (full adjustment), and 3) a propensity score weighted, simple (unadjusted) version. Table S1 displays the weights used to correct for non-participation bias due to age and education. We used logistic regression analysis to estimate the probability of participation in this study based on age and education (full study sample N=146). Data from the whole Nor-COAST sample was used. The inverse of the probability of participation constituted the weights. Highly educated participants in the adult age group had the highest probability of participation in this study and hence were assigned the lowest weight (3.45), while the oldest participants with low education were underrepresented and hence assigned the highest weight (10.23). Primary education was defined as <11 years, secondary 11 to 12 years, and tertiary and tertiary 13+ years of education.

**Table S1 Inverse probability weights**

| Age | Education | Weight |
| --- | --- | --- |
|  | Primary | 4.66 |
| Adult age (<65 years) | Secondary | 3.99 |
|  | Tertiary | 3.45 |
|  | Primary | 5.99 |
| Young old age (65 to 74 years) | Secondary | 5.07 |
|  | Tertiary | 4.33 |
|  | Primary | 7.79 |
| Old age (75 to 84 years) | Secondary | 6.54 |
|  | Tertiary | 5.53 |
|  | Primary | 10.23 |
| Old-old age (85+ years) | Secondary | 8.54 |
|  | Tertiary | 7.17 |

**Table S2 Structural brain MRI features without and with inverse probability weighting**

| MRI measurement | Time since the incident stroke | | | | | |
| --- | --- | --- | --- | --- | --- | --- |
| without IPW | N | Baseline | N | 18 months | N | 36 months |
| Cortex volume (mL) | 115 | 440.26 (45.71) | 135 | 433.69 (48.31) | 81 | 430.28 (43.67) |
| Thalamus volume (mL) | 115 | 13.08 (1.77) | 135 | 12.66 (1.71) | 81 | 12.43 (1.54) |
| Hippocampus volume (mL) | 115 | 7.19 (1.10) | 135 | 7.20 (1.08) | 81 | 7.14 (1.09) |
| BAG (y) | 114 | -0.40 (9.45) | 135 | -0.74 (9.69) | 81 | -2.37 (9.80) |
| with IPW |  |  |  |  |  |  |
| Cortex volume (mL) | 115 | 435.24 (44.68) | 135 | 427.56 (46.90) | 81 | 426.16 (44.47) |
| Thalamus volume (mL) | 115 | 12.84 (1.80) | 135 | 12.39 (1.67) | 81 | 12.21 (1.49) |
| Hippocampus volume (mL) | 115 | 7.03 (1.11) | 135 | 7.00 (1.06) | 81 | 6.97 (1.12) |
| BAG (y) | 114 | -0.45 (9.35) | 135 | -0.94 (9.57) | 81 | -2.54 (9.69) |

Abbreviations: Inverse probability weighting (IPW), Brain age gap (BAG)

**Table S3 Primary exposure variable without and with inverse probability weighting**

| Accelerometric estimate | Time since incident stroke | | | | | |
| --- | --- | --- | --- | --- | --- | --- |
| without IPW | N | 3 months | N | 18 months | N | 36 months |
| Average daily steps count | 133 | 3220 (1984) | 142 | 2565 (1583) | 94 | 2970 (1751) |
| with IPW |  |  |  |  |  |  |
| Average daily steps count | 133 | 2975 (1860) | 142 | 2363 (1500) | 94 | 2768 (1708) |

Abbreviations: Inverse probability weighting (IPW)

**Figure S2 Time-dependent threshold of the maximum effect between step count and thalamus volume**

This figure shows the curvilinear association, including the time interaction depicted in Table 2. It shows the predicted margins of thalamus volume between 4100 and 5700 steps to visualize the associated maximum effect of steps at 18 (blue curve and blue shaded 95% confidence band) and 36 (red curve and red shaded 95% confidence band) months post-stroke.

Regression Coefficient

95%CI

Full model

Simple model

Full weighted model

Simple weighted model

**A**

**B**

**C**

**D**

**Figure S3 LME model comparison of the association between average daily step count and structural brain MRI features post-stroke.**

Full model refers to LME models as presented in the main manuscript, adjusted for sex, age, education, time of measurement, and NIHSS. For the volumetric analysis, we additionally adjusted for MRI scanner and total estimated intracranial volume. Simple models were not adjusted for scanner factors. Weighted LMEs were conducted using the propensity score weights. Graphs **A** to **C** display the estimated regression coefficients with 95% Confidence Intervals of the simple (unadjusted) LMEs, simple weighted LMEs, and the full (adjusted) weighted LMEs for comparison against the full models (in blue). In **D,** due to the curvilinear association between average daily step count and thalamus volume, we present the full range of average daily step count observed in the study population against predicted thalamus volume for each model configuration (full model in blue). The Confidence Intervals for the estimated coefficients of all 4 model configurations across **A**, **B, & C** overlap, none including zero.

| BAG | Cortex volume | Hippocampus volume | Thalamus volume |
| --- | --- | --- | --- |
| 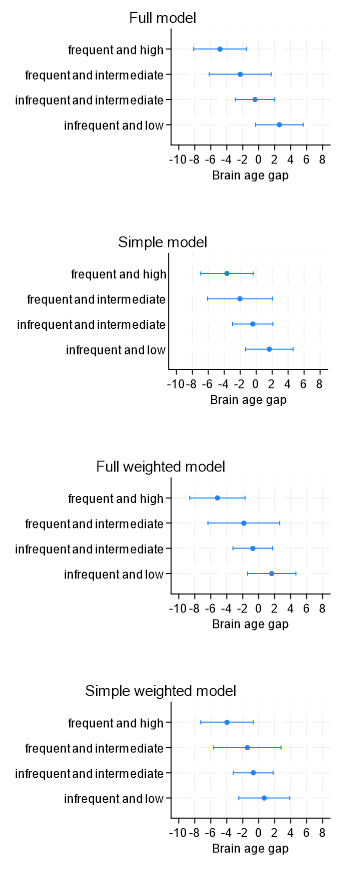 | 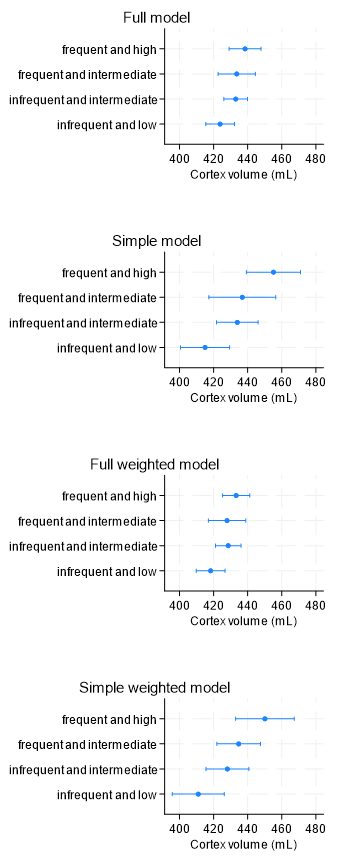 |  |  |

**Figure S4 Model comparison** **of the association between PA trajectory group membership and structural brain MRI features post-stroke**

Full model refers to linear mixed effect models (LME) adjusted for sex, age, education, time of measurement, and stroke severity. For the volumetric analysis, we additionally adjusted for MRI scanner and total estimated intracranial volume. Simple models were not confounder adjusted. Weighted LMEs were conducted using the propensity score weights. The model comparison of the association between PA trajectory group membership and structural brain MRI features post-stroke indicates results to be robust against weighting and adjustment. The pattern of the predicted outcomes and the 95% CI indicated by the blue dot and lines, respectively, suggest a stepwise association between PA trajectory groups and brain age gap (BAG), cortex, hippocampus, and thalamus volumes.

**Table S4 Characteristics of included versus excluded participants**

|  | Unweighted | | | | | Weighted |
| --- | --- | --- | --- | --- | --- | --- |
|  | **Nor-COAST**  N=815 | **Included**  N=146 | | **Excluded**  N=669 | | **Included**  N=146 |
| Female, n (%)  Male, n (%) | 366 (44.9)  449 (55.1) | 0 | 60 (45.7)  86 (58.9) | 0 | 306 (45.7)  363 (54.3) | 43.1%  56.9% |
| Age, years mean (SD) | 73.53 (11.75) | 0 | 70.28 (11.14) | 0 | 74.24 (11.74) | 72.93 (10.48) |
| Living alone  Living with someone  Living in institution | 298 (36.6)  508 (62.3)  9 (1.1) | 0 | 47 (32.2)  99 (67.8) | 0 | 251 (37.5)  409 (61.1)  9 (1.35) | 36.2%  63.8%  0% |
| Education, y., mean (SD) | 11.97 (3.73) | 0 | 12.90 (3.85) | 0 | 11.77 (3.68) | 12.23 (3.83) |
| Smoking status, n (%)  Never  Smoker  Ex-smoker  Unknown | 346 (42.6)  145 (17.9)  317 (39.0)  4 (0.5) | 0 | 68 (46.6)  25 (17.1)  53 (36.3)  0 | 3 | 278 (41.7)  120 (18.0)  264 (39.6)  4 (0.6) | 43.6%  17.1%  39.3%  0 |
| BMI, mean (SD) | 25.95 (4.21) | 4 | 26.38 (4.18) | 62 | 25.86 (4.22) | 26.19 (0.35) |
| TOAST, n (%)  Arteriosclerosis  Cardiac emboli  Small vessel disease  Other  Unknown | 77 (10.8)  173 (24.3)  147 (20.6)  22 (3.1)  294 (41.2) | 11 | 12 (8.9)  37 (27.4)  34 (25.2)  2 (1.5)  50 (37.0) | 91 | 65 (11.3)  136 (23.5)  113 (19.6)  20 (3.5)  244 (42.2) | 8.6%  28.4%  24.6%  1.2%  37.3% |
| NIHSS score, day 1  mean (SD)  median [perc^25-75^] | 3.58 (5.04)  2 [1-4] | 0 | 2.83 (4.69)  2 [0-3] | 23 | 3.75 (5.11)  2 [1-5] | 2.87 (4.47)  2 [0-4] |
| pre-stroke mRS, n (%)  0  1  2  3  4  5 | 378 (46.4)  196 (24.1)  133 (16.3)  79 (9.7)  27 (3.3)  2 (0.3) | 0 | 80 (54.8)  44 (30.1)  19 (13.0)  3 (2.1)  0  0 | 0 | 298 (44.5)  152 (22.7)  114 (17.0)  76 (11.4)  27 (4.0)  2 (0.3) | 51.8%  31.5%  14.3%  2.4%  0  0 |
| **3-months follow-up** | | | | | | |
| mRS, n (%)  0  1  2  3  4  5 | 100 (14.3)  219 (31.29)  209 (29.9)  88 (12.6)  62 (8.9)  22 (3.1) | 1 | 25 (17.2)  54 (37.2)  54 (37.2)  9 (6.2)  3 (2.1)  0 | 114 | 75 (13.5)  165 (29.7)  155 (27.9)  79 (14.2)  59 (10.6)  22 (4.0) | 17.2%  36.9%  35.7%  8.2%  2.0% |
| MoCA  mean (SD)  median [perc^25-75^] | 24.15 (4.69)  25 [22-27] | 4 | 25.63 (3.14)  26 [24-28] | 229 | 23.67 (5.01)  25 [21-27] | 25.19 (3.28)  26 [23 – 28] |
| SPPB,  mean (SD)  median [perc^25-75^] | 9.23 (3.35)  11 [8-12] | 5 | 10.11 (2.41)  11 [9 to 12] | 228 | 8.95 (3.55)  10 [7-12] | 9.76 (2.49)  10 [8-12] |
| HADS total  mean (SD)  median [perc^25-75^] | 7.53 (6.41)  6 [3-11] | 15 | 7.08 (5.98)  6 [2-10] | 248 | 7.67 (6.54)  6 [3-11] | 7.48 (6.08)  6 [3-11] |
| FSS-7,3 months  mean (SD)  median [perc^25-75^] | 3.33 (1.84)  3.29 [1.57-4.86] | 11 | 3.06 (1.80)  2.57 [1.29-4.43] | 244 | 3.42 (1.84)  3.43 [1.57-4.86] | 3.19 (1.85)  2.71 [1.57-4.71] |

Note: The weighting successfully removed selection bias in age, education, and NIHSS. We decided against including NIHSS in the logistic regression model to create the weights partly due to the simpler model removing the differences and partly because the score was quite skewed when including NIHSS. The same was true when including pre-stroke mRS.

Abbreviations: Body Mass Index (BMI), Trial of Org 10172 in Acute Stroke Treatment (TOAST), modified Rankin Scale (mRS), Montreal Cognitive Assessment (MoCA), Short Physical Performance Battery (SPPB), 7-item Fatigue Severity Scale (FSS-7)

**Table S5** Distribution of MRI data across PA trajectory groups

| Time of measurement | Included  total | Infrequent  and low | Infrequent and intermediate | Frequent and intermediate | Frequent and high |
| --- | --- | --- | --- | --- | --- |
| N | 146 | 39 (26.7) | 54 (37.0) | 21 (14.4) | 32 (21.9) |
| MRI observations at 18 months n (%) | 135 (92.5) | 36 (92.3) | 51 (94.4) | 18 (85.7) | 30 (93.8) |
| MRI observations at 36 months n (%) | 81 (55.5) | 19 (48.7) | 32 (59.3) | 14 (66.7) | 16 (50.0) |

In total 146 participants contributed to this study with MRI data from 18- and 36-month follow-ups. We demonstrate that both the distribution by group size and attrition were roughly equal across the differing PA trajectory groups.

**Table S6** Estimated margins of BAG, cortex, hippocampus, and thalamus volume across PA trajectory groups

| Trajectory group | BAG (in mL) | Cortex in (mL) | Hippocampus in (mL) | Thalamus in (mL) |
| --- | --- | --- | --- | --- |
| Infrequent and low | 2.59 (1.52) | 423.86 (4.29) | 6.81 (0.13) | 12.09 (0.19) |
| Infrequent and intermediate | -0.46 (1.25) | 432. 98 (3.55) | 7.23 (0.12) | 12.47 (0.16) |
| Frequent and intermediate | -2.31 (1.98) | 433.60 (5.59) | 7.12 (0.17) | 12.92 (0.25) |
| Frequent and high | -4.84 (1.69) | 438.47 (4.76) | 7.34 (0.15) | 12.99 (0.21) |

Estimated margins are based on the LME models as specified in the main article. Hence, the above margin means of brain age gap (BAG) are adjusted for sex, age, education, stroke severity, and time of measurement. The LMEs for the volumetric estimates (cortex, hippocampus, and thalamus) included intracranial volume and MRI scanner as additional covariates. Note that hippocampus volume and thalamus volume are presented as the sum of both ipsi-and contralesional hemispheres. The PA trajectories are based on longitudinal accelerometer data from 3, 18, and 36 months poststroke across light, moderate PA, and the number of sit-to-stand transitions. They may therefore be interpreted as phenotypes of longitudinal PA behavior post-stroke.

**Figure S5** Kernel Density Plots: Included versus excluded MRI outcomes from baseline

**Table S7** Physical activity trajectory groups and structural brain MRI features at 36 months post-stroke (n=81)

| **BAG, years** | Coefficient | Std. Err. | p-value | 95%CI | |
| --- | --- | --- | --- | --- | --- |
| PA trajectory  Ref.  Infrequent and low  Infrequent and intermediate  Frequent and intermediate | 7.71  6.04  3.26 | 3.15  2.90  3.35 | 0.02  0.04  0.33 | 1.44 to 13.97  0.26 to 11.82  -3.42 to 9.95 | |
| **Cortex volume (mL)** |  |  |  |  |  |
| PA trajectory  Ref.  Infrequent and low  Infrequent and intermediate  Frequent and intermediate | -11.06  -5.86  -4.59 | 8.49  7.76  8.99 | 0.20  0.45  0.61 | -27.99 to 5.87  -21.34 to 9.62  -22.52 to 13.34 | |
| **Hippocampus volume (mL)** |  |  |  |  | |
| PA trajectory  Ref.  Infrequent and low  Infrequent and intermediate  Frequent and intermediate | -0.71  -0.22  -0.32 | 0.31  0.28  0.33 | 0.03  0.45  0.33 | -1.33 to -0.09  -0.78 to 0.35  -0.98 to 0.33 | |
| **Thalamus volume (mL)** |  |  |  |  | |
| PA trajectory  Ref.  Infrequent and low  Infrequent and intermediate  Frequent and intermediate | -1.23  -0.82  -0.48 | 0.41  0.38  0.43 | 0.004  0.03  0.28 | -2.05 to -0.41  -1.57 to -0.07  -1.34 to 0.39 | |

To change time lags, we entered physical activity (PA) trajectory group membership as an explanatory constant variable and brain MRI features from 36 months as outcome variables. The “frequent and high” group is used as a reference group. Estimates are adjusted for sex, age, education, time of measurement, and stroke severity (NIHSS-1). We adjusted for the MRI scanner and total estimated intracranial volume in volumetric analysis. The hippocampus and thalamus represent the sum of estimated volumes of the left and right ventricles, accordingly.

Abbreviations: Brain age gap (BAG), milliliter (mL).

Nagin, D. S. (2014). "Group-Based Trajectory Modeling: An Overview." Annals of Nutrition and Metabolism **65**(2-3): 205-210.
